# “It's a cause I believe in”: factors motivating participation and engagement in longitudinal, respiratory-focused research studies

**DOI:** 10.1186/s12890-023-02582-6

**Published:** 2023-08-04

**Authors:** Andrew J. Synn, Katherine E. Menson, Mercedes R. Carnethon, Ravi Kalhan, Elizabeth A. Sugar, George R. Washko, Robert A. Wise, Michelle N. Eakin

**Affiliations:** 1 Division of Pulmonary, Critical Care, and Sleep Medicine, Beth Israel Deaconess Medical Center, 330 Brookline Ave, KSB-23, Boston, MA 02215 USA; 2 Division of Pulmonary and Critical Care Medicine, University of Vermont, Burlington, VT USA; 3 Department of Preventive Medicine, Feinberg School of Medicine, Northwestern University, Chicago, IL USA; 4 Division of Pulmonary and Critical Care Medicine, Northwestern University, Chicago, IL USA; 5 Department of Biostatistics, Johns Hopkins Bloomberg School of Public Health, Baltimore, MD USA; 6 Division of Pulmonary and Critical Care Medicine, Brigham and Women’s Hospital, Boston, MA USA; 7 Division of Pulmonary and Critical Care Medicine, Johns Hopkins University, Baltimore, MD USA

**Keywords:** Qualitative methods, Young adults, Research study participation

## Abstract

**Background:**

Key to the success of any prospective cohort study is the effective recruitment and retention of participants, but the specific factors that influence younger adults of the Millennial generation to participate in research are not well-understood. The objective of this qualitative study was to identify factors that motivated participation and engagement in longitudinal research studies focused on respiratory health among a diverse group of young adults.

**Methods:**

We conducted qualitative, semi-structured interviews with 50 younger adult participants (aged 25–35 years) regarding factors influencing their participation in longitudinal research studies. Thematic analysis was used to develop, organize, and tabulate the frequency of key themes. In exploratory analyses, we examined for patterns in the distribution of key themes across racial, ethnic, or socioeconomic groups.

**Results:**

Participants identified several key themes that affected their willingness to participate in longitudinal studies. These included the health-related benefits generated by research (both to the individual and to society at-large), factors related to the institution and study team conducting the research, concerns regarding unethical and/or unrepresentative study design, and barriers to participation in research. Certain factors may be more impactful to underrepresented groups, including concerns regarding data privacy and confidentiality.

**Conclusions:**

In this diverse group of younger adults, we identified specific factors that motivated participation and predicted high engagement in longitudinal research studies focused on respiratory health. Implementing and integrating these factors into study protocols may improve recruitment and retention, including among participants who are historically underrepresented in research.

**Supplementary Information:**

The online version contains supplementary material available at 10.1186/s12890-023-02582-6.

## Background

Chronic lung disease, including conditions such as COPD, asthma, and interstitial lung disease, are a major cause of global morbidity and mortality [1]. While a number of prospective, long-term cohort studies have successfully elucidated risk factors for the onset and progression of cardiovascular disease [2, 3], no longitudinal cohort study has been dedicated to identifying the determinants of peak lung function among healthy, younger adults of the Millennial generation. The American Lung Association (ALA) Lung Health Cohort study (https://www.lung.org/research/lung-health-cohort-study) is the first national cohort to focus on the long-term respiratory health of younger adults.

A key factor in the success of any longitudinal cohort study is recruitment and retention over the study period. Inadequate recruitment and retention results in underpowered studies and higher risk for type II error [4], leading to wasted resources and invalid scientific results. Ideally, the study cohort should be representative of the general population. Minority populations are disproportionately affected by barriers to study participation, and historically have been underrepresented in research studies, leading to inequities in the applicability of research findings to already disadvantaged groups [5, 6]. A number of studies have identified factors such as transportation, limited understanding of the clinical trial process, and privacy concerns as barriers to recruitment in historically underrepresented populations [7]. While recruitment barriers do not significantly differ between urban and rural populations, patients of lower socioeconomic status are more likely to be skeptical of clinical trials [8, 9]. More recent publications have suggested that prior recruitment strategies may not be as relevant or effective for today’s young adults [10, 11].

Previous meta-analyses have identified strategies to maximize successful retention in clinical trials, including effective culturally competent communication, flexible study protocols (including scheduling and location), and material and health-related informational benefits [12]. Specifically, involvement of family members in the study process, emphasizing community benefits, and study flexibility is highlighted as a facilitator to retention in Asian communities [13]. Similar benefits of study retention in longitudinal research were found by making 2 or more successful phone calls to Alaska Native and American Indian people, rather than contact by text or email [14]. While prior studies have identified that young adults respond favorably to incentives as a method of retention, the methods of contact and follow-up are dated in the era of the internet and social media [15]. Additionally, it is unclear whether these prior findings are relevant to participation in longitudinal studies focused on respiratory health, in which success is dependent on routine testing and imaging over an extended period of time. Consequently, the newly formed ALA Lung Health Cohort study requires the application of effective recruitment strategies to attract the diverse, younger populations of interest.

The ALA Lung Health Cohort study will aim to recruit a diverse sample of 4,000 younger adults (aged 25–35 years, with an explicit focus on representation across racial, ethnic, and socioeconomic groups) in order to monitor lung health over time. To help maximize recruitment efficacy for this study, we sought to understand factors that affect participation and engagement in a group of younger adults and identify potential recruitment/retention strategies.

## Methods

### Study population

We recruited a purposive convenience sample of 50 participants using email and social media postings sent on behalf of the ALA, and from an existing database of Johns Hopkins research study participants. Participants were selected by purposive sampling and invited to contact the study team directly or provide their contact information via survey. No participants were previously known to the study team.

Given our goal to analyze perspectives by thematic analysis from individuals matching the ALA Lung Health Cohort study’s recruitment profile, we included all adults between 25–35 years of age and attempted to recruit a sample that was roughly balanced by sex and three categories of educational attainment (high school education or less, some college, and college degree or higher). Those with existing severe lung disease (> 4 respiratory-related hospitalizations in the prior 12 months), current pregnancy/breastfeeding, history of cancer (other than non-melanoma skin cancer), cardiovascular disease, and those who were unable to read/understand English or provide consent were excluded. Our methods followed the consolidated criteria for reporting qualitative research (COREQ) [16].

All participants provided oral informed consent via telephone. Participants who consented and completed a phone interview received $25 in compensation for their participation. The study protocol was approved by the Johns Hopkins Institutional Review Board.

### Study design

Information on gender, age, marital status, race/ethnicity, highest attained level of education, occupation, annual income, and employment status were collected for descriptive analysis. Using an IRB-approved interview guide (see Supplemental Materials), we conducted semi-structured phone interviews to understand potential strategies for recruiting and retaining younger adults into longitudinal cohort studies, such as the ALA Lung Health Cohort study. The interview guide was internally pilot-tested with 2 research staff matching the inclusion criteria of the study. Interviews were conducted by a masters-level trained qualitative interviewer who was unknown to the participants (TB) under the supervision of a clinical psychologist and qualitative researcher (MNE). Interviews were conducted using Zoom (San Jose, CA, USA) video conferencing software or via telephone. Each participant was interviewed once. Interview questions were modified during subsequent interviews using an iterative process to ensure that a broad representation of information was obtained. Interviews were conducted until thematic saturation was achieved, which was defined as no emergence of novel themes over a period of 3 consecutive interviews. Interviews were audio-recorded and transcribed by TranscribeMe (Oakland, CA, USA) for analysis. One participant refused recording of interview, and therefore the interview was documented by notetaking. No other field notes were taken. No participants redacted their interview. Participant data were de-identified prior to analysis.

### Data analysis

Analysis of transcripts were performed by study investigators who were not involved in conducting the interviews. Themes were independently generated by a review of the transcripts by multiple investigators (JA and MNE) using open, inductive coding to generate the codebook [17]. Two independent coders (JA and MK) used the codebook to subsequently code all of the transcripts using an iterative process to add additional themes when identified. Coder agreement was examined using percent agreement and Cohen’s kappa statistic. Any discrepancies were adjudicated by the PI (MNE) using a consensus-based approach to ensure consistency in thematic coding. Thematic analysis was used to develop, organize, and categorize key themes based on similarity [18]. Transcripts were coded and analyzed using NVivo 11.0 software (QSR International, Melbourne, Australia). Transcripts were not returned to participants, and feedback was not requested on findings. Given a specific interest in identifying recruitment strategies that were germane to populations historically underrepresented in research, we examined for patterns in the distribution of key themes across racial, ethnic, or socioeconomic groups.

## Results

Overall, 133 emails were sent out. Of those, 37 individuals were unable to be contacted, 12 were not interested, 25 were not eligible and 59 consented to participate in the study. Reasons for ineligibility included: age (*n* = 14, 56%), breastfeeding/pregnant (*n* = 4, 16%), history of cancer (*n* = 3, 12%) history of cardiovascular disease (*n* = 2, 8%) current ALA employee (*n* = 1, 4%) or respiratory condition other than asthma (*n* = 1, 4%). Of those individuals who consented, 5 individuals withdrew prior to completing the interview, 4 were unable to be contacted, and 50 completed the interview and comprised the final sample. Characteristics of the study population are displayed in Table 1. Agreement between the two coders was high (mean percent agreement = 99.6 ± 1.0, Cohen’s kappa = 0.80). Mean length of interviews was 40.0 ± 11.2 min. The results of our thematic analysis revealed certain domains that appeared to be of particular importance for predicting high study engagement in a longitudinal cohort study, including perceived health-related value of study results, the institution/team conducting the study, and research study design.

**Table 1:** Study sample characteristics (*n* = 50)

Demographic factor	Mean ± SD or n (%)
Age (years)	31.3 ± 3.0
Female	36 (72)
Self-Reported Race	
White (W)	33 (66)
Black (B)	10 (20)
Asian or Pacific Islander (API)	4 (8)
Multiple	3 (6)
Ethnicity	
Non-Hispanic or Latino	47 (94)
Hispanic or Latino	1 (2)
Did Not Disclose	2 (4)
Marital Status	
Married	23 (46)
Divorced/separated	6 (12)
Never married	21 (42)
Educational status	
High school education or less	5 (10)
Some college or Associate degree	11 (22)
Bachelor degree or higher	32 (64)
Did Not Disclose	2 (4)
Employment status	
Part-time	7 (14)
Full-time	38 (76)
Not employed or disabled	5 (10)
Income category	
< $50,000/year	23 (46)
$50,000 – $75,000/year	16 (32)
> $75,000/year	11 (22)

### Perceived health-related benefits

A consistent theme that emerged from participant interviews was the perceived health and scientific value generated by research (Table 2). Participants reported that they were more likely to engage in scientific research if it appeared to benefit their own health or the health of their relatives. Multiple participants (31yo API,M; 31yo W,F; 34yo W,F) noted their interest in participating in research was grounded in their “personal connection” to the population being studied. One participant (27yo W,M) stated that he/she would “happily [participate in a research study for lung disease] to show my support for my loved one with lung disease.” However, we also identified a strong theme related to the broader value of research to societal health (i.e. to improve the health of individuals outside of the participants’ direct circle), including the impact of research on laws and policies governing public health, and general altruistic factors. As two younger adult participants stated, they wanted to participate in research “if it’s a cause that I believe in” (31yo W,M) and “something that fits me to my core” (28yo W,F).

**Table 2:** Perceived health-related value of study results

Qualitative Theme	Exemplar Quote
Personal value: one’s own health	[interested in research that informs] “how to take care of myself better. What can I do to improve my overall health.” – 34yo W,F “I'd be more than happy to know how this modern world [such as climate change] is affecting my health.” – 31yo W,M
Personal value: relatives/family members	“If a family member had lung disease, and they wanted people to participate in a research study for lung disease, I would happily do that to show my support for the loved one.” – 27yo W,M
Societal value: Broad scientific value to the community	“people live longer now than they used to because…we've eradicated a lot of diseases and illnesses and sicknesses through research.” – 31yo W,M “I really feel like research being done really brings a lot of hope…of promising new discoveries [to impact the overall health of society].” – 32yo W,F
Societal value: Inform health-related policy	“[research can inform] policy change…that helps the general health of everybody.” – 28yo W,F “If someone didn't research something or try to attempt something, we wouldn't be as civilized as we are now.” – 28yo W,F
Societal value: Altruism	“my overall want for doing research [is to]…benefit others who are going through…lung health issues…what can I do to help benefit other people? It's not about benefiting myself.” – 34yo W,F

*Abbreviations*: *API* Asian or Pacific Islander, *B* Black, *W* White, *F* Female, *M* Male

### Institution and study team

Participants described a number of factors related to the institution and team of investigators conducting the study that affected their motivation to participate (Table 3). The following themes were identified around how the larger institution and environment support the study: the general reputation of the institution; the perception that their study-related data were handled appropriately in accordance with existing standards; and, a sense that the institution had a stake in the local community in which the study was being conducted. Regarding specific institutional factors, one participant (32yo W,F) stated that in order to be motivated to participate in research, “I need to feel like whoever was funding the research, that it was somebody I could really rely on.” In our exploratory sub-group analyses, we found that this theme of perceived institutional trust and integrity as a positive factor in research participation was similarly represented across racial, ethnic, and socioeconomic lines. In contrast, we identified a possible difference based on race regarding factors related to data privacy and confidentiality, with Black participants reporting more concerns as compared with White participants. For example, two participants, both Black women, reported concerns about whether their research data would “be kept confidential,” and that it “would be alarming” if the study team did not explicitly review procedures for data protection. On the other hand, White participants generally reported little to no concerns, with one respondent stating “I have no issues with that. I see no fear.” Finally, themes related to specific study team attributes were also identified as being important to participants, including a sense that the team was welcoming, responsive, willing to accommodate the needs of research participants, and representative of the local community. One participant (34yo W,F) noted that it helps when the study team “just recognizes that it is a commitment…[and] makes it as easy and as flexible for [me] as possible.”

**Table 3:** Institution and study team factors influencing research study participation

Qualitative Theme	Exemplar Quote
Institutional factor: Integrity	“[less interest in] studies funded by certain companies or industries that might be a bit skewed, just based off of who is sponsoring them…[those] researchers don't necessarily want to say negative things because that's who's sponsoring and paying for their study.” – 33yo W,F
Institutional factor: Privacy and confidentiality	“What are they doing with [my research data]…is it going to be kept confidential?” – 26yo B,F
Institutional factor: Trusted reputation and local engagement	“I'd [want to hear about research studies] from a reliable source like…my work…[or] a community group that I'm involved with…just having the feeling that someone vetted it before they sent it to me.” – 32yo W,F
Study-team specific factor: Welcoming, accommodating, and communicative	“[it would motivate participation if] the research team…could explain to me what the study is about.” – 34yo W,F “If I have to go digging and it’s not clear off the bat I’d be less likely to participate.” – W,F (age undisclosed) “[I would] like to be updated on the results.” – 30yo W,M “would be interesting to see how I helped form the study.” – 29yo W,F
Study-team specific factor: diversity of investigative team	“I would like to see diversity in the research team and the cohort.” – 30yo API/B,M
Study-team specific factor: Sense of belonging	“it'd be cool to be a part of a participant group…and be a part of that little community. People are always wanting to kind of be involved in a community.” – 28yo W,F “[I would be more likely to join a study if] I could do it with a friend…so we can talk to each other about our experiences as we’re doing it…that process [gives you] more support.” – 31yo W,F “[I would be more likely to participate] if I had friends that were taking part in a research study.” – 26yo W,M

### Research study design

Understanding participants’ concerns regarding both general and specific research procedures were identified as being a key component affecting motivation to participate in prospective studies (Table 4). In terms of the general conduct of research, participants described a number of overlapping concerns including those related to inefficient, poorly designed, unethical, fraudulent, and/or unrepresentative research as major barriers to participate. One participant (32yo W,F) stated that his/her primary concern was about research ethics: “the first thing is integrity, knowing whether or not people are being honest, if they're doing it for the right reasons, or if they been vetted enough, [and] if the information has been gathered properly and tallied properly.” Regarding study-specific facilitators, several themes were identified including the major effect of convenience and efficiency of study visits, and the potential risks of study interventions. As one participant (30yo W,M) noted, an immediate barrier “prevent[ing] me from participating in a research study…would be if there's anything [about the study protocol/procedures] that would negatively impact me.” In sub-group analyses, we identified that inconvenience of study visit has a potentially disproportionate impact on lower income groups. While participants of White, Black, and Asian/Pacific Islander race all reported that convenience was a highly important factor in their willingness to participate in research, the vast majority of these responses were from lower- and middle-income groups who were employed full-time. In contrast, among participants who reported that monetary compensation was an important factor motivating study participation, we found no apparent patterns in race, employment status, or income; for example, several respondents in the highest income categories reported that they would be unlikely to participate in a study without some form of financial incentive.

**Table 4:** Research design-related factors influencing research study participation

Qualitative Theme	Exemplar Quote
General research concern: Inefficient, poorly designed, or unrepresentative research	“A research study might have cost a lot of money to try to learn something, and maybe they didn't learn as much as they really wanted to out of it.” – 30yo W,M “[the results of many research studies] are not very representative…they’re usually done on predominantly white, middle-aged populations, and do not transfer across groups very well. So representation is really important.” –31yo API,F
General research concern: Fraudulent research	“One [concern] that comes to mind right now is the whole thing about vaccines causing autism, and how that was clearly manipulated data…even though it was proven wrong later, people are still caught up on it.” – 31yo W,F
Study-specific concern: Study-related risks	“if someone offered me to, say, "Hey, take this pill and see if it works for you. We don't know what it will do but we'll see if it works." That's something I would not entertain because it might have harmful effects on my body.” – 31yo Hispanic W,M “I think anything that would be too invasive or would maybe give me pause… if you're talking any type of invasive procedure, is maybe something that is going to require overnight hospital stay, or like you said, a CT that might require contrast and needles and things like that, that may be a deterrent.” – 35yo B,F
Study-specific concern: Convenience of study visits	“I just wouldn't really have tons of time during the weekday to physically go somewhere.” – 29yo W,F
Study-specific concern: Efficiency of study visits	“Obviously, just the least amount of information that can be asked each time, making sure it's streamlined, no time is being wasted just sitting, making sure that once the participant shows up, their time is being used very wisely as much as possible…like when I go to a regular doctor, I sit there sometimes for a half-hour or an hour, just waiting to see the doctor for five minutes.” – 30yo W,M “if we were to have appointments or scheduling for anything, I think it would be more comfortable if things are going on schedule and not to have huge wait times.” – 33yo W,F
Study-specific concern: Compensation for travel	“I'm not asking to get paid a bunch of money or get rich off of something. I just want to feel like it's fair. Okay, I have to take off work. If I get paid to be off of work for my job, I'm not worried about it. [COMPANY] is still paying me. But if I have to drive to [CITY NAME], I'd expect my mileage to be paid for. I'm not trying to get rich or make money off the deal. I want to feel like it's not costing me something.” – 31yo W,M
Study-specific concern: Compensation for time	“Financial compensation helps.” – 29yo W,F “Being compensated for my time.” – 31yo W,M “I feel like if I'm going to use my time to help others, I know that that sounds really weird, like I'm helping others but I want to get paid. That just sounds very selfish. But at the same time, during a pandemic I'm like, ‘Oh, I need money.’” – 27yo API/W,F

### Retention

Finally, we examined themes related to participant-specific factors influencing longitudinal retention and ongoing engagement in a prospective cohort study (Table 5). A major theme that emerged was that a study that could generate a sense of community, belonging, and value among its participants was more likely to retain engagement over time. For example, one participant (34yo API,M) noted that “creating an idea of ownership” would provide a personal stake for the participants to continue their participation, while another (31yo API,M) felt that study participation would rekindle an “altruistic…part of themselves” to promote their ongoing engagement in the study. This theme appeared to resonate particularly strongly with participants of Black and Asian/Pacific Islander race. In addition, participants emphasized the importance of regular communication by the study team as a key factor to improve retention, although the preferred methods for follow-up differed between participants in our sample and included phone calls, text messaging, and social media-based contact.

**Table 5:** Strategies to influence participant retention in longitudinal studies

Qualitative Theme	Exemplar Quote
Sense of meaning and investment	“It would be really fun to create some camaraderie and community among the participants that are doing the research… it would potentially increase and prolong engagement from your participants.” – 34yo API,M “create an idea of ownership within the study itself could be really exciting for participants like, ‘I have some stake in this somehow.’” – 34yo API,M “foster some community between the folks who are all participating so that the participants don't feel like they're by themselves.” – 28yo B,F “Maybe I'm kind of a dreamer here but sort of get people to remember the altruistic part of their life, a part of themselves that said, ‘Oh yeah, I have a duty, and I said I would participate in this, which helps people with lung problems.’” – 31yo API,M “More likely to do it [participate in research studies] when they know, ‘Oh, okay. This is going to help my community because of these three reasons.’ Or, ‘This is going to help me for these three reasons.’” – 27yo API/W,F
Ongoing communication	“I'd say just communication over time…maybe it's an email update every quarter or every other month, or just something that keeps it on the radar and not forgotten about.” – W,F (age undisclosed) “Gives them an easy way to stay involved and feel involved and not forget about the study.” – 31yo W,M “[I appreciate the study team] texting you little messages with just a prompt, like, "Hey, Miss [NAME]. Thanks for being involved in our study. You have helped us to uncover XYZ for such-and-such such." – 35yo B,F
Preferred method of follow-up contact	“I get hundreds of emails a day, so they could be overlooked…I think it's easier to talk over the phone. Things could be misinterpreted through text or through email.” – 35yo B,F “You can almost text at any point in time. You don't have to take time away by being on the phone. To me, it's easier than email.” – 31yo W,F “I can respond to a text when I have time. I can get on social media when I have time.” – 26yo W,F

## Discussion

In this study, we identified specific factors that motivated participation in a research study focused on respiratory health among a diverse population, specifically of younger adults. The results of our thematic analyses revealed that factors in certain domains predicted high study engagement among younger adults for longitudinal studies, including the perceived health-related value of research study results, the institution/team conducting the study, and research study design.

Longitudinal studies, by nature, are at risk of attrition given the ongoing time investment required by participants. Lack of motivation in both staff and participants is one of the greatest threats to the success of prospective studies, regardless of the subject under study [6]. However, specific factors that predict engagement in longitudinal studies in today’s younger adult population are not well described in the literature, and in particular, little is known about what types of recruitment strategies will optimally reduce barriers to participation and improve access to research among historically underrepresented groups – a critical need highlighted in a recent ATS research statement [5].

Our findings showed that a near-universal motivator of research study participation in young adults was to gain access to health-related benefits – whether for themselves, their loved ones, and/or for the greater good. These findings are largely consistent with the existing literature [19], but our analysis emphasized that participants are also highly motivated to contribute health-related value to others, with no expectation of self-benefit. Emphasizing altruistic factors during recruitment may appeal to broader populations (particularly among groups that historically have had disproportionately limited access to the benefits of biomedical research) and improve participant engagement and commitment across the study period.

Institutional factors (including integrity, privacy, institutional reputation, a welcoming staff, and a sense of community) were a second domain that motivated ongoing participation in longitudinal studies among our participants. These findings are also generally supported by results of previous studies, which note that well-organized, persistent, friendly, and culturally sensitive communicators are aspects of successful studies (although often not incorporated into protocols) [20], while burdensome enrollment processes can disproportionately affect minority groups [5]. These findings highlight the role of implicit bias training and clinical competency for study staff in reducing barriers to research participation [21]. Similarly, a lack of transparency, fear and mistrust of the study, and ambiguity in the purpose of the research study contribute to poor recruitment, while building relationships with both patients and communities has been associated with successful clinical trials [22]. Older adults have demonstrated more trust in medical research compared to younger adults, however age is not as strong of a predictor of trust compared to race, ethnicity, primary spoken language, and disability status [23]. Our participants specifically noted an interest in how research data are collected, processed, and stored, suggesting that improved communication protocols regarding these study specific procedures may enhance trust and therefore improve retention, a finding that to our knowledge has not been described before. Emphasizing transparency and purpose of the study with recruitment information may have more impact for this age range. Notably, our study was performed at the beginning of the COVID-19 pandemic, a setting in which public interest in research methods (and how the results of research studies may affect personal and public health) was heightened.

Although our study was not designed to definitively address differential research attitudes along racial and socioeconomic lines, our exploratory subgroup analyses demonstrated that certain factors may be more impactful to underrepresented groups. We found that, while the integrity of research team members was important across all subgroups, concerns regarding data privacy and confidentiality were particularly significant to racial minorities, although we caution that these are exploratory analyses and require validation in larger cohorts. This is similar to the results of a recent study which found that Black and Native American participants expressed positive sentiments generally about the potential benefits of precision medicine research, but were hesitant to participate due to privacy concerns, the potential for information to be used in a discriminatory manner, and the concern that findings would not be used to benefit their communities given the history of prior abuses in research [24]. In context, our findings continue to highlight the essential need to maintaining trust, transparency, and clarity regarding study-related procedures, especially for certain historically disadvantaged groups who have previously suffered direct harm from unethical research experiments [25].

Our analyses demonstrated that certain study design elements were important to motivating research participation. Compensation for time spent engaged in research was universally cited as a positive factor by participants across the income spectrum. The importance of compensating for research participation is well-described in prior studies, and ethical standards dictate that compensation to research participants should be based on local wages and paid in full, regardless of study completion, to prevent concealing side effects or adverse events [26]. Similarly, monetary incentives were shown to improve completion rates for questionnaires compared to non-monetary material incentives [27], with one meta-analysis demonstrating that response rate more than doubled when a monetary incentive was offered [28]. We also found that full-time employed participants in lower income brackets were much more sensitive to the convenience of study visits and the flexibility of study procedures compared to those with higher incomes. This may represent a specific area to improve the recruitment of less advantaged socioeconomic subgroups, and our results highlight the need to consider location and scheduling flexibility of study site(s), transportation options, and childcare as strategies to promote recruitment for under-represented populations.

Finally, our results demonstrated that certain communication techniques may be more relevant for recruitment of younger adults. Participants in our study tended to prefer technology-based communication, including personalized texts, emails, and utilization of online social media networks, to maintain interest and feel valued in their contributions. This is consistent with prior studies demonstrating that younger adults are most likely to be responsive to initial contact by mobile phone [29], and that social media was more effective than mailings, news media, or partner communications among younger women [11] (an effect which may extend to traditionally harder-to-reach groups [30]). Given the rapidly changing nature of technology, it is imperative that recruitment efforts be mindful of newly developed and trending platforms. Relatedly, our study found that strategies to create a sense of meaning and community about the research study may help to improve recruitment and retention. This theme resonated strongly across all subgroups in our study, and this sense of investment and community may be particularly impactful to racial minorities [21].

Our study has a number of limitations. Our sample size is relatively small and we used non-probability methods to select our sample, as is common for qualitative research. Participants were recruited from databases maintained by organizations with strong ties to research (Johns Hopkins, the American Lung Association), which may have affected the range of perspectives we observed. While our goal was to elicit responses from a group of younger adults that was roughly balanced by sex and three categories of educational attainment, our sample consisted predominantly of women and those who completed a college education. These factors may limit the generalizability of our findings to other populations, particularly regarding our exploratory subgroup analyses given the design of our study and the small size of these subgroups.

Our findings of this qualitative study add to the literature regarding attitudes towards research participation in younger adults, which remains poorly described. Given growing scientific interest in the interception of at-risk individuals before severe disease has developed, the recruitment and retention of younger individuals is likely to become an increasingly important focus in future research studies. Our findings, which demonstrate certain factors that appear to be particularly important to our sample of younger adults, may therefore be helpful to investigators planning longitudinal studies, particularly those with a respiratory focus.

For example, the results of this qualitative study informed multiple aspects of the study design and recruitment strategy for the ALA Lung Health Cohort. Based on the qualitative findings we developed our study branding and messaging to focus on community. Our study logo includes the line “BeLUNG to something bigger,” to provide a sense of belonging and appealing to altruism. Our full recruitment strategy includes a social media campaign targeting our demographics with these messaging to link potential participants with local recruitment sites. We have created and disseminated videos and blogs using participant experiences to describe benefits of research. These testimonials have been shown on local media news stations to reach individuals who may not traditionally engage with research. We also have engaged local ALA chapters or primary care offices as trusted organizations to link potential participants with research sites.

We also significantly modified the study design to include more remote procedures including remote completion of online surveys, pulmonary function tests and other data collection strategies to limit the time participants had to be physically present in the clinic. We ensure that during consent and enrollment our staff are well trained to be welcoming and accommodating while taking the time to address questions and review privacy concerns.

## Conclusions

In this qualitative study of younger adults, we found that certain domains appeared to influence participation in longitudinal respiratory-focused research, including health-related study benefits, institutional factors, study methods/design, and communication strategies. Implementing and integrating these factors into study protocols may improve recruitment and retention, including among participants who are historically underrepresented in research.

### Supplementary Information

**Additional file 1.**

## Data Availability

The datasets used and/or analyzed during the current study are available from the corresponding author on reasonable request.

## Abbreviations

ALA: American Lung Association
API: Asian-Pacific Islander
B: Black
COREQ: Consolidated Criteria for Reporting Qualitative Research
F: Female
M: Male
W: White

## References

[1] Viegi G, Maio S, Fasola S, Baldacci S (2020). global burden of chronic respiratory diseases. J Aerosol Med Pulm Drug Deliv.

[2] Mahmood SS, Levy D, Vasan RS, Wang TJ (2014). The framingham heart study and the epidemiology of cardiovascular disease: a historical perspective. Lancet.

[3] Berry JD, Dyer A, Cai X (2012). Lifetime risks of cardiovascular disease. N Engl J Med.

[4] Fogel DB (2018). Factors associated with clinical trials that fail and opportunities for improving the likelihood of success: a review. Contemp Clin Trials Commun.

[5] Thakur N, Holguin F, Alvidrez J (2021). Enhancing recruitment and retention of minority populations for clinical research in pulmonary, critical care, and sleep medicine an official american thoracic society research statement. Am J Respir Crit Care Med.

[6] Briel M, Speich B, Von EE, Gloy V (2019). Comparison of randomized controlled trials discontinued or revised for poor recruitment and completed trials with the same research question: a matched qualitative study. Trials.

[7] Cunningham-Erves J, Joosten Y, Kusnoor SV (2023). A community-informed recruitment plan template to increase recruitment of racial and ethnic groups historically excluded and underrepresented in clinical research. Contemp Clin Trials.

[8] Friedman DB, Foster C, Bergeron CD, Tanner A, Kim SH (2015). A qualitative study of recruitment barriers, motivators, and community-based strategies for increasing clinical trials participation among rural and urban populations. Am J Heal Promot.

[9] Clark LT, Watkins L, Piña IL (2019). Increasing diversity in clinical trials: overcoming critical barriers. Curr Probl Cardiol.

[10] Rabin C, Horowitz S, Marcus B (2013). Recruiting young adult cancer survivors for behavioral research. J Clin Psychol Med Settings.

[11] Wasfi R, Stephens ZP, Sones M (2021). Recruiting participants for population health intervention research: Effectiveness and costs of recruitment methods for a cohort study. J Med Internet Res.

[12] Robinson KA, Dinglas VD, Sukrithan V (2015). Updated systematic review identifies substantial number of retention strategies: Using more strategies retains more study participants. J Clin Epidemiol.

[13] Singh P, Ens T, Hayden KA (2018). Retention of ethnic participants in longitudinal studies. J Immigr Minor Heal.

[14] Mills DE, Schaefer KR, Beans JA (2022). Retention in a 6-Month smoking cessation study among Alaska Native and American Indian People. Am Indian Alaska Nativ Ment Heal Res.

[15] Booker CL, Harding S, Benzeval M (2011). A systematic review of the effect of retention methods in population-based cohort studies. BMC Public Health.

[16] Tong A, Sainsbury P, Craig J (2007). Consolidated criteria for reporting qualitative research (COREQ): A 32-item checklist for interviews and focus groups. Int J Qual Heal Care.

[17] Hsieh HF, Shannon SE (2005). Three approaches to qualitative content analysis. Qual Health Res.

[18] Vaismoradi M, Turunen H, Bondas T (2013). Content analysis and thematic analysis: Implications for conducting a qualitative descriptive study. Nurs Heal Sci.

[19] Sheridan R, Martin-Kerry J, Hudson J, Parker A, Bower P, Knapp P (2020). Why do patients take part in research? An overview of systematic reviews of psychosocial barriers and facilitators. Trials.

[20] Abshire M, Dinglas VD, Cajita MIA, Eakin MN, Needham DM, Himmelfarb CD (2017). Participant retention practices in longitudinal clinical research studies with high retention rates. BMC Med Res Methodol.

[21] Haley SJ, Southwick LE, Parikh NS, Rivera J, Farrar-Edwards D, Boden-Albala B (2017). Barriers and strategies for recruitment of racial and ethnic minorities: perspectives from neurological clinical research coordinators. J Racial Ethn Heal Disparities.

[22] Natale P, Saglimbene V, Ruospo M (2021). Transparency, trust and minimizing burden to increase recruitment and recruitment in trials: a systematic review. J Clin Epidemiol.

[23] Smirnoff M, Wilets I, Ragin DF (2018). A paradigm for understanding trust and mistrust in medical research: The Community VOICES study. AJOB Empir Bioeth.

[24] Rosas LG, Nasrallah C, Park VT (2020). Perspectives on precision health among racial/ethnic minority communities and the physicians that serve them. Ethn Dis.

[25] Alsan M, Wanamaker M (2018). Tuskegee and the health of black men. Q J Econ.

[26] Anderson EE (2019). A proposal for fair compensation for research participants. Am J Bioeth.

[27] Brueton VC, Tierney JF, Stenning S (2014). Strategies to improve retention in randomised trials: a Cochrane systematic review and meta-analysis. BMJ Open.

[28] David MC, Ware RS (2014). Meta-analysis of randomized controlled trials supports the use of incentives for inducing response to electronic health surveys. J Clin Epidemiol.

[29] Lingham G, Mackey DA, Seed N (2020). Re-engaging an inactive cohort of young adults: Evaluating recruitment for the Kidskin Young Adult Myopia Study. BMC Med Res Methodol.

[30] Whitaker C, Stevelink S, Fear N (2017). The use of facebook in recruiting participants for health research purposes: a systematic review. J Med Internet Res.

