# Supplementary material for: “It's a cause I believe in”: factors motivating participation and engagement in longitudinal, respiratory-focused research studies
Source: BMC Pulm Med. 2023 Aug 4;23:285. doi: 10.1186/s12890-023-02582-6 (PMC10401812; doi:10.1186/s12890-023-02582-6)
Supplement: Supplementary file 1 — Additional file 1. [file 12890_2023_2582_MOESM1_ESM.pdf]

## SUPPLEMENTARY MATERIALS:

Interview Guide: Qualitative assessment of recruitment and retention strategies for a lung health cohort study

Experiences with research

1. What if any experience do you have with clinical research?
    a. IF YES
        i. Tell me about that experience
        ii. What did you like about being in a research study?
        iii. What did you not like about being in a research study?
    b. IF NO
        i. What prompted/made you want to join this study?
        ii. What do you think would have made you less likely to join?
        iii. What do you think are the reasons that you never joined a research study before this one?
            1. Why haven't you looked into it before?
            2. Have you ever wanted to participate in a research study?

Attitudes toward research

1. How do you think research may impact your current health? How about your health in the future? (probe- what specifically?)
2. How do you think research may impact society's health overall?
3. What are some positive things you think about when you hear about research studies in the news or from people you know?
4. What are some negative things you think about when you hear about research studies in the news or from people you know?

Reasons for participating

1. If you were invited to join a research study, what are some of the reasons you would agree or not agree to participate?
    a. What would motivate you to join?
    b. What do you think would help you to participate in the study?
2. What, if anything, concerns you about your health (right now)?
    a. What, if any, role do you see research has in addressing those concerns?
3. What concerns you about the health of people your age? Or family and friends?
    a. Why is that a concern for you?
    b. What, if any, role do you see research has in addressing these concerns?

Barriers to participating

1. What are some things that may prevent you from joining research studies?
    a. Are there any things the research team could do to help overcome those issues/barriers to your participation?

There are many types of research. Some that are focused on testing a new medicine in people who have a specific disease. Other research studies look at people before they are sick to see if they can identify

what may cause a disease. We have plans to begin enrolling for a research study that would be looking at healthy adults and follow them over a long period of time so that we can learn more about lung health and how those problems might develop. These visits will take up to 4 hours and would include a physician examination, surveys, blood draws, lung function testing and a CT scan.

Benefits of research

1. What, if anything, do you see as a potential benefit for participating in a lung health study like this?
2. What if anything does not interest you about this study? How could we make it more comfortable for participants?
3. What, if anything, do you see would be a benefit for research focused on healthy individuals?
4. What would make you more or less likely to join a study looking at healthy people? (Probe- why that?)

Retention practices

So, in addition to having enough people participate in this large lung health research study. Our goal is also to maintain regular contact with our participants so that we can see how lung health changes over time.

1. How do you keep in touch with people from the past?
2. If you were in a research study what would be the best ways to keep in touch?
3. What ideas if any do you have for keeping people engaged in a research study?
